# Mechanism-Driven Metabolic Engineering for Bio-Based Production of Free *R*-Lipoic Acid in *Saccharomyces cerevisiae* Mitochondria

**DOI:** 10.3389/fbioe.2020.00965

**Published:** 2020-08-20

**Authors:** Binbin Chen, Jee Loon Foo, Hua Ling, Matthew Wook Chang

**Affiliations:** ^1^Department of Biochemistry, Yong Loo Lin School of Medicine, National University of Singapore, Singapore, Singapore; ^2^NUS Synthetic Biology for Clinical and Technological Innovation (SynCTI), National University of Singapore, Singapore, Singapore

**Keywords:** lipoic acid, *Saccharomyces cerevisiae*, metabolic engineering, lipoamidase, *de novo* biosynthesis

## Abstract

Lipoic acid is a valuable organosulfur compound used as an antioxidant for dietary supplementation, and potentially anti-diabetic and anti-cancer. Currently, lipoic acid is obtained mainly through chemical synthesis, which requires toxic reagents and organic solvents, thus causing environmental issues. Moreover, chemically synthesized lipoic acid is conventionally a racemic mixture. To obtain enantiomerically pure *R*-lipoic acid, which has superior bioactivity than the *S* form, chiral resolution and asymmetric synthesis methods require additional reagents and solvents, and often lead to wastage of *S*-lipoic acid or precursors with undesired chirality. Toward sustainable production of *R*-lipoic acid, we aim to develop a synthetic biology-based method using engineered yeast. Here, we deepened mechanistic understanding of lipoic acid biosynthesis and protein lipoylation in the model yeast *Saccharomyces cerevisiae* to facilitate metabolic engineering of the microbe for producing free *R*-lipoic acid. In brief, we studied the biosynthesis and confirmed the availability of protein-bound lipoate in yeast cells through LC-MS/MS. We then characterized *in vitro* the activity of a lipoamidase from *Enterococcus faecalis* for releasing free *R*-lipoic acid from lipoate-modified yeast proteins. Overexpression of the lipoamidase in yeast mitochondria enabled *de novo* free *R*-lipoic acid production in vivo. By overexpressing pathway enzymes and regenerating the cofactor, the production titer was increased ∼2.9-fold. This study represents the first report of free *R*-lipoic acid biosynthesis in *S. cerevisiae*. We envision that these results could provide insights into lipoic acid biosynthesis in eukaryotic cells and drive development of sustainable *R*-lipoic acid production.

## Introduction

Lipoic acid is an essential cofactor required for several key enzymes involved in aerobic metabolism and the glycine cleavage system in most organisms (Cronan et al., 2005; Cronan, 2016). It can be used as an antioxidant for dietary supplementation due to its ability to bind directly or indirectly with free radicals (Croce et al., 2003). Furthermore, findings from clinical trials have shown that lipoic acid can increases insulin sensitivity, which supports its application as an anti-diabetic drug (Lee et al., 2005). Lipoic acid has also shown to inhibit the proliferation of breast tumor cells, and hence demonstrates its potential application as an anti-cancer drug (Li et al., 2015). Currently, lipoic acid is obtained mainly through chemical synthesis processes, which conventionally generates equal amounts of the two enantiomeric *R* and *S* forms of lipoic acid (Balkenhohl and Paust, 1999; Ide et al., 2013). However, in biological systems, lipoic acid exists solely in the *R* form; *S*-lipoic acid is a by-product during chemical synthesis. Therefore, *R*-lipoic acid in general shows bioactivity superior to *S*-lipoic acid, and in some cases, *S*-lipoic acid is detrimental to health. For example, *R*-lipoic acid was shown to protect the lens in eyes from forming cataract, while *S*-lipoic acid showed the reverse effect by potentiating deterioration of the lens (Kilic et al., 1995). Thus, it is beneficial to obtain *R*-lipoic acid in the enantiomerically pure form to maximize the health effects of lipoic acid and prevent potential side effects caused by *S*-lipoic acid. Yet, chiral separation and asymmetric synthesis methods used to attain pure *R*-lipoic acid lead to wastage of the *S* form of lipoic acid or precursors of undesired chirality (Blaschke et al., 1994; Villani et al., 2003, 2005; Purude et al., 2015), hence reducing the efficiency of resource utilization in synthesizing the compound. Moreover, compared to racemic lipoic acid synthesis, these procedures for preparing pure *R*-lipoic acid lengthen the production process, and require additional reagents and solvents, which incur higher manufacturing costs and greater impact on the environment. In view that chemical synthesis of *R*-lipoic acid also involves toxic reagents and catalysts, and entails many steps, biological engineering of microbial cell factories for production of free *R*-lipoic acid presents an attractive avenue for obtaining enantiomerically pure *R*-lipoic acid in a sustainable and environmentally-friendly manner. Bacterial production of lipoic acid through metabolic engineering has been shown in bacteria, including *Escherichia coli*, *Pseudomonas reptilivora*, *Listeria monocytogenes, and Bacillus subtilis* (Ji et al., 2008; Moon et al., 2009; Christensen et al., 2011; Storm and Müller, 2012; Sun et al., 2017). The lipoic acid biosynthesis and protein lipoylation pathways are most well-studied in *E. coli* over the past two decades. There are two complementary pathways for lipoic acid biosynthesis and protein lipoylation in *E. coli*: (i) *de novo* biosynthesis pathway where endogenous octanoic acid is attached to apo-proteins by LipB, followed by sulfur insertion by LipA, and (ii) scavenging pathway where exogenous lipoic acid or octanoic acid is transferred to unlipoylated apo-form of proteins by LplA (Sun et al., 2017). Compared to bacteria, *Saccharomyces cerevisiae*, a model yeast strain, offers a number of advantages for biochemical production due to its inherent abilities to withstand lower temperature, pH changes and phage attack (Chen et al., 2015; Jin et al., 2016; Foo et al., 2017). Importantly, unlike *E. coli*, yeast lacks a lipoic acid scavenging pathway that binds free lipoic acid to proteins via an ATP- and energy-expending process (Booker, 2004). Hence, *S. cerevisiae* inherently does not consume free lipoic acid, which is a beneficial characteristic that allows accumulation of our target compound, i.e., free *R*-lipoic acid. Therefore, we aim to investigate *S. cerevisiae* as a production host for free *R*-lipoic acid biosynthesis. Hereafter, lipoic acid specifically refers to *R*-lipoic acid.

To engineer *S. cerevisiae* for free lipoic acid biosynthesis, it is of utmost importance to understand the formation process of lipoate-bound proteins. In yeast, there are three well-known lipoate-dependent enzyme systems: glycine cleavage system (GCV), α-ketoglutarate dehydrogenase (KGDC) and pyruvate dehydrogenase (PDH) (Schonauer et al., 2009). GCV is involved in the cleavage of glycine to ammonia and C1 units, which is essential for utilization of glycine as a sole source of nitrogen (Sinclair and Dawes, 1995; Piper et al., 2002). KGDC catalyzes the oxidative decarboxylation of 2-oxoglutarate to succinyl-CoA, a precursor of several amino acids and the source of succinate, the entry point to the respiratory chain (Repetto and Tzagoloff, 1991). PDH catalyzes the oxidative decarboxylation of pyruvate, thereby linking cytosolic glycolysis and mitochondrial respiration (Boubekeur et al., 1999). Gcv3p, Kgd2p, and Lat1p are the lipoate-bound subunits of GCV, KGDC, and PDH, respectively (Nagarajan and Storms, 1997). To form lipoylated Gcv3p, Kgd2p, and Lat1p, a two-step conversion has been hypothesized for lipoic acid synthesis and protein attachment in yeast mitochondria (Hermes and Cronan, 2013). Lip2p and Lip3p have been demonstrated to encode octanoyltransferases that utilize octanoyl-ACP or octanoyl-CoA to attach an octanoyl group to the apo-form of lipoate-dependent proteins (Stuart et al., 1997; Marvin et al., 2001; Hermes and Cronan, 2013). A lipoyl synthase Lip5p then catalyzes the insertion of two sulfurs into the octanoate carbon chain (Sulo and Martin, 1993). Ultimately, lipoic acid is formed and bound to Gcv3p, Kgd2p, and Lat1p via an amide linkage between its carboxyl group and the epsilon amino group of a lysine residue of the proteins (Sulo and Martin, 1993). Interestingly, it has been discovered that Lip2p and Lip5p are required for lipoylation of all three proteins while Lip3p is required for lipoylation of Kgd2p and Lat1p but not Gcv3p (Hermes and Cronan, 2013). Despite the aforementioned reports, lipoic acid synthesis and attachment to target proteins have not been thoroughly studied and still not well-understood in yeast. To facilitate our efforts to produce free lipoic acid in *S. cerevisiae*, more investigation into the formation of lipoylated protein was needed.

To release free lipoic acid from lipoate-bound proteins, an amidase is essential for hydrolysis of the amide bond between lipoic acid and the lipoylated proteins. Lipoamidase isolated from *Enterococcus faecalis* (EfLPA) is a member of the Ser-Ser-Lys family of amidohydrolases (Jiang and Cronan, 2005). This enzyme has been demonstrated to liberate free lipoic acid from lipoic acid-bound H protein of GCV, and E2 subunit of KGDC and PDH from *E. coli* (Spalding and Prigge, 2009). While functional heterologous expression of EfLPA has been demonstrated in bacterial hosts, the activity of EfLPA in yeast has to be evaluated to ascertain the suitability of the enzyme for application toward free lipoic acid production in *S. cerevisiae*.

**FIGURE 1:**
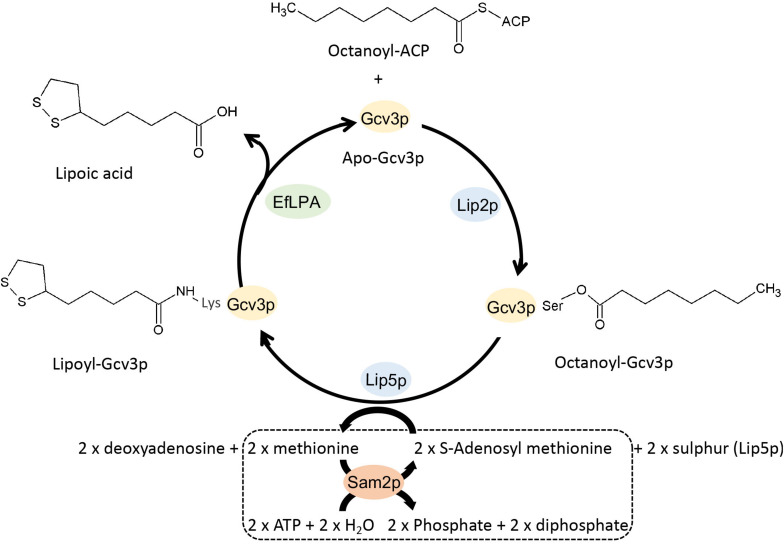
Schematic view of the metabolic pathway for the production of lipoic acid in engineered *S. cerevisiae*. Apo-Gcv3p is a substrate protein while octanoyl and lipoyl-Gcv3p are the two intermediates in the lipoic acid producing pathway. Lipoyl-Gcv3p is the lipoic acid bound subunit of glycine cleavage system (GCV). Lip2p and Lip5p work as catalyst enzymes. EfLPA is the cleaving enzyme for releasing lipoic acid. Sam2p is a cofactor regeneration enzyme required to regenerate the S-adenosyl methionine cofactor, as shown in the dotted box. Lip2p, octanoyltransferase; Lip5p, lipoyl synthase; EfLPA, lipoamidase from *E. faecalis*; Sam2p, S-adenosylmethionine synthase 2. All reactions are in mitochondria.

Herein, we aimed to better our understanding of lipoic acid biosynthesis, protein lipoylation and EfLPA activity to aid the engineering of *S. cerevisiae* for producing free lipoic acid. Concurrently, we employed metabolic engineering strategies to improve lipoic acid production. To this end, we first confirmed the availability of lipoate-bound proteins in yeast and characterized them through liquid chromatography-tandem mass spectrometry (LC-MS/MS). We then determined the *in vitro* activity of EfLPA to validate its functional expression and to select a suitable lipoylated protein as the target substrate for EfLPA. To develop a free lipoic acid-producing strain, we modified EfLPA for translocation to the mitochondria, where lipoylated proteins reside. Finally, to enhance the lipoic acid production, the selected substrate protein (i.e., Gcv3p), catalytic enzymes (i.e., Lip2p, and Lip5p), and cofactor regenerating enzymes (i.e., Sam1p and Sam2p) were overexpressed (Figure 1). The proteomic analysis, enzyme characterization and metabolic engineering approaches collectively enabled unprecedented free lipoic acid production in *S. cerevisiae* to be accomplished and the titer to be further boosted. Importantly, this study demonstrated the first functional expression of a lipoamidase in *S. cerevisiae* and its application for releasing free lipoic acid from a protein of yeast origin. We envisage that the approaches described here will provide insights into lipoic acid biosynthesis in yeast and advance metabolic engineering of yeast chassis for bioproduction of lipoic acid.

## Materials and Methods

### Strains and Media

*E. coli* TOP10 (Invitrogen) and Luria-Bertani (Becton, Dickinson and Company) were used for cloning experiments unless otherwise stated. 100 mg/L ampicillin was used for selection of positive colonies where applicable. The yeast strain *S. cerevisiae* BY4741 (ATCC) was used for genetic engineering for lipoic acid production.

*S. cerevisiae* BY4741 wild-type and mutant strains were cultured in rich medium YPD/YPGR (1% yeast extract, 2% peptone, and 2% D-glucose or 2% galactose with 1% raffinose), synthetic minimal medium lacking uracil SC-U (0.67% yeast nitrogen base, 0.192% uracil dropout and 2% D-glucose), medium lacking lysine SC-L (0.67% yeast nitrogen base, 0.18% lysine dropout and 2% D-glucose), medium lacking leucine SC-LE (0.67% yeast nitrogen base, 0.16% leucine dropout and 2% D-glucose), or medium lacking both leucine and uracil SC-LU (0.67% yeast nitrogen base, 0.154% leucine and uracil dropout, and 2% D-glucose). 2% agar was supplemented for making solid media. Yeast growth media components were purchased from Sigma-Aldrich, MP Biomedicals and BD (Becton, Dickinson and Company). 5-Fluoroorotic acid (5-FOA, Fermentas) or geneticin (G418, PAA Laboratories) was used for selection. Cysteine (0.2 mg/ml) and ferrous sulfate (0.2 mg/ml) (Sigma-Aldrich) were supplemented into growth culture where necessary. Yeast cells were cultivated at 30^*o*^C in flasks and shaken at 225 rpm.

### Plasmid Construction and Gene Integration

*EfLPA* gene (GenBank Accession No. AY735444) was codon-optimized for *S. cerevisiae* and synthesized by Integrated DNA Technologies. *EfLPA* genes with and without mitochondrial targeting peptide (MTP) sequence were ligated between P_GAL1_ promoter and T_CYC1_ terminator, which were amplified from the *S. cerevisiae* genomic DNA. *EfLPA* expression cassettes with and without MTP were inserted to the vector pRS41K (Euroscarf), resulting in plasmids pRS41K-P_GAL1_-mEfLPA-T_CYC1_ and pRS41K-P_GAL1_-EfLPA-T_CYC1_, respectively. The plasmids pRS41K-P_GAL1_-mEGFP-T_CYC1_ and pRS41K-P_GAL1_-EGFP-T_CYC1_ were, similarly, constructed for *EGFP* with and without MTP, respectively. The constructed recombinant plasmids are listed in Table 1. The list of primers used was shown in Supplementary Table S1.

**TABLE 1:** Strains and plasmids used in this study.

Strains or plasmids	Description	Source
**Strains**		
*E. coli* Top10	F^–^ *mcrA Δ(mrr-hsdRMS-mcrBC)*φ80*lacZΔ*M15 *ΔlacX74 recA1 araD*139 *Δ(ara-leu)*7697 *galU galK rpsL(Str^*R*^) endA1 nupG*	Invitrogen
***S. cerevisiae***		
BY4741	MATa *his3Δ1 leu2Δ0 met15Δ0 ura3Δ0*	ATCC
BY4741-GCV3	BY4741 with P_TEF1_-GCV3-T_CYC1_ (lys2 site)	This study
BY4741-LAT1	BY4741 with P_TEF1_-LAT1-T_ADH1_ (lys2 site)	This study
BY4741-KGD2	BY4741 with P_TEF1_-KGD2-T_KGD2_ (lys2 site)	This study
BY4741-control	BY4741 with plasmid pRS41K	This study
BY4741-EfLPA	BY4741 with plasmid pRS41K-P_GAL1_-EfLPA-T_CYC1_	This study
BY4741-mEfLPA	BY4741 with plasmid pRS41K-P_GAL1_-mEfLPA-T_CYC1_	This study
BY4741-EGFP	BY4741 with plasmid pRS41K-P_GAL1_-EGFP-T_CYC1_	This study
BY4741-mEGFP	BY4741 with plasmid pRS41K-P_GAL1_-mEGFP-T_CYC1_	This study
BY4741-GCV3-mEfLPA	BY4741 with P_TEF1_-GCV3-T_CYC1_ (lys2 site) and plasmid pRS41K-P_GAL1_-mEfLPA-T_CYC1_	This study
BY4741-GCV3-LIP2-LIP5-mEfLPA	BY4741 with P_TEF1_-GCV3-T_CYC1_ (lys2 site), P_TEF1_-LIP2-T_LIP2_ (CS6 site), P_PGI1_-LIP5-T_LIP5_ (CS6 site) and plasmid pRS41K-P_GAL1_-mEfLPA-T_CYC1_	This study
BY4741-GCV3-LIP2-LIP5-mSAM1-mEfLPA	BY4741 with P_TEF1_-GCV3-T_CYC1_ (lys2 site), P_TEF1_-LIP2-T_LIP2_ (CS6 site), P_PGI1_-LIP5-T_LIP5_ (CS6 site), P_ADH1_-mSAM1-T_SAM1_ (CS8) and plasmid pRS41K-P_GAL1_-mEfLPA-T_CYC1_	This study
BY4741-GCV3-LIP2-LIP5-mSAM2-mEfLPA	BY4741 with P_TEF1_-GCV3-T_CYC1_ (lys2 site), P_TEF1_-LIP2-T_LIP2_ (CS6 site), P_PGI1_-LIP5-T_LIP5_ (CS6 site), P_ADH1_-mSAM2-T_SAM2_ (CS8) and plasmid pRS41K-P_GAL1_-mEfLPA-T_CYC1_	This study
**Plasmids**		
pIS385	AmpR, URA3	Euroscarf
pRS41K	ARS/CEN origin, kanMX	Euroscarf
pRS41K-P_GAL1_-EfLPA-T_CYC1_	pRS41K carrying EfLPA under P_GAL1_ control	This study
pRS41K-P_GAL1_-mEfLPA-T_CYC1_	pRS41K carrying MTP-EfLPA under P_GAL1_ control	This study
pRS41K-P_GAL1_-EGFP-T_CYC1_	pRS41K carrying EGFP under P_GAL1_ control	This study
pRS41K-P_GAL1_-mEGFP-T_CYC1_	pRS41K carrying MTP-EGFP under P_GAL1_ control	This study

Chromosomal integration of the expression cassettes P_TEF1_-GCV3-T_CYC1_, P_TEF1_-KGD2-T_KGD2_ and P_TEF1_-LAT1-T_ADH1_ into the *LYS2* site were conducted based on the method previously described by Sadowski et al. (2007), where the integrative vector pIS385 (Euroscarf) containing URA3 selectable marker was used for integration. In addition, the cassettes P_TEF1_-LIP2-T_LIP2_ and P_PGI1_-LIP5-T_LIP5_ were integrated into intergenic site CS6 while P_ADH1_-mSAM1-T_SAM1_ and P_ADH1_-mSAM2-T_SAM2_ were integrated into intergenic site CS8 (Xia et al., 2017) based on Clustered Regularly Interspaced Short Palindromic Repeats (CRISPR) and CRISPR-associated (Cas) system previously established (Dicarlo et al., 2013). To clone *GCV3*, *LAT1*, *KGD2*, *LIP2*, *LIP5*, *SAM1*, and *SAM2*, genomic DNA of *S. cerevisiae* was used as the PCR template. All proteins abovementioned were located to the mitochondria through its native MTP (for Gcv3p, Lat1p, and Kgd2p) or MTP from yeast cytochrome c oxidase subunit IV (COX4) (for mEfLPA, mSam1p, and mSam2p) (Maarse et al., 1984). Hexa-histidine tag was added to either the C- or N-terminus of these proteins for expression analysis. Oligonucleotide primers used are listed in Supplementary Table S1.

### Detection of Lipoylated and Octanoylated Proteins

Cells were pre-cultured in 5 ml YPD medium overnight and then diluted in 100 ml YPD medium using 500 ml flask to achieve an initial OD_600_ of 0.4. After growth for 18 h, cells were harvested by centrifugation. Cell pellets were re-suspended in 25 ml lysis buffer (0.3 M NaCl, 50 mM sodium phosphate, pH 6.5). Cells were lysed with a high-pressure homogenizer (EmulsiFlex-C3, AVESTIN, Inc.) at 25,000 psi. The soluble cell lysate was collected by centrifugation and mixed an equal volume of 8 M Guanidine hydrochloride. 300 μl final products were injected into Agilent 1260 Infinity binary HPLC (Agilent). The proteins were resolved with an mRP-C18 High-Recovery Protein column (Agilent) at a solvent flow rate of 1.5 ml/min and column temperature of 80^*o*^C. The mobile phases A and B were 0.1% trifluoroacetic acid/water and 0.1% trifluoroacetic acid/acetonitrile, respectively. The proteins were eluted with the following gradient: 0–1 min (10–30% B), 1–12 min (30–50% B), 12–13 min (50–80% B), 13–14 min (80% B), 14–15 min (80–10% B), and 15–17 min (10% B). Protein collection started from 1 min and 12 successive 1 min fractions were collected. The proteins were dried overnight in a Speedvac concentrator (Thermo Fisher Scientific). Each fraction of proteins was re-suspended with 50 μl 0.5 M triethylammonium bicarbonate with 1 μg Glu-C (Promega). The mixture was incubated overnight.

Seven microliter digested peptides was loaded into Agilent 1260 infinity HPLC-Chip/MS System (Agilent) equipped with a PortID-Chip-43 (II) column (Agilent). A linear gradient of acetonitrile was used to elute the peptides from the HPLC-Chip system at a consistent flow rate of 0.35 μl/min. For LC separation, 0.2% formic acid/water (mobile phase A) and 0.2% formic acid/acetonitrile (mobile phase B) were used. The samples were eluted with the following gradient through a nano pump: 0–1 min (7–10% B), 1–35 min (10–30% B), 35–37 min (30–80% B), 37–38 min (80% B), 38–40 min (80–7% B), and 40–43 min (7% B). The eluted samples were directly infused into a mass spectrometer for detection. The mass spectra were scanned in the range of 100–1600 m/z with a scan rate of 3 spectra per second. The MS/MS scan range is 80–2000 m/z with a scan rate of 4 spectra per second. Mass data was collected in positive ion mode at a fragmentor voltage of 175 V and skimmer voltage of 65 V.

### Peptide Post-translational Modification (PTM) Analysis

The SPIDER feature of PEAKS 8 software (Bioinformatics Solutions Inc., Waterloo, Canada) (Zhang et al., 2012) was used to identify the peptides with PTMs based on mass difference. The yeast peptides were searched with the following search parameters. The precursor mass error tolerance was 100 ppm (part-per-million) while the fragment mass error tolerance was 0.1 Da. The fixed PTM was carbamidomethylation (C) (+57.02) and variable PTMs were lipoyl (K) (+188.03), octanoyl (TS) (+126.10), oxidation (M) (+15.99) and oxidation (HW) (+15.99). The peptide and protein identification reliability score (−10lgP, where P is the probability of identification) was set at a threshold of 15 and 20, respectively, corresponding to confident identifications. The database used was UniProtKB/Swiss-Prot^1^.

### Protein Modeling for Structure Visualization

SWISS-MODEL^2^ (Waterhouse et al., 2018) was used to build the 3D structure models of Gcv3p, Kgd2p and Lat1p proteins from their amino acid sequences using homology modeling techniques. The structures were predicted based on templates available in the SWISS-MODEL template library (SMTL) which aggregates information of experimental structures from Protein Data Bank (PDB). PyMOL Molecular Graphics System (Schrödinger, Inc., New York, United States) (Schrodinger, 2015) was used to observe the structures.

Template homolog proteins with 41, 37, and 48% sequence identity were used for modeling of Gcv3p, Kgd2p, and Lat1p, respectively. The template protein for Gcv3p is glycine cleavage system protein H from *Mycobacterium tuberculosis* (PDB chain id: 3hgb.1.A), while for Kgd2p, and Lat1p, only the N-termini (lipoyl domains) were modeled due to the lack of templates with crystal structure of full length. The template for the N-terminus (lipoyl domain) of Kgd2p is the lipoyl domain of E2 component of 2-oxoglutarate dehydrogenase complex in *Azotobacter vinelandii* (PDB chain id: 1ghj.1.A). The N-terminus of Lat1p (lipoyl domain) was modeled using the dihydrolipoyllysine-residue acetyltransferase component of the pyruvate dehydrogenase complex in *Homo sapiens* (PDB chain id: 1y8n.1.B).

### Protein Overexpression and Purification

Cells were pre-cultured in 5 ml medium overnight and then diluted in 50 ml induction medium using 200 ml flask to achieve an initial OD_600_ of 0.4. After overnight cell growth, the yeast cells were harvested by centrifugation. The cell pellets were re-suspended in lysis buffer (0.5 M NaCl, 20 mM sodium phosphate, 20 mM imidazole, pH 6.8) and lysed with a high pressure homogenizer (EmulsiFlex-C3, AVESTIN, Inc.) at 25,000 psi. After centrifugation, the insoluble protein and cell debris were separated from the soluble protein. To check protein expression, the soluble protein was boiled with Laemmli sample buffer (Bio-Rad) and separated on an SDS-polyacrylamide gel. The proteins in the gels were transferred onto western blotting membrane and using HRP conjugated anti-6x His-tag antibody (Thermo Fisher Scientific) as described previously (Chen et al., 2013). To detect protein expressed in the mitochondria, mitochondrial proteins were extracted using yeast mitochondria isolation kit (Biovision). The extracted proteins will be boiled with Laemmli sample buffer and detected through western blotting as described.

To purify the proteins, the soluble proteins were incubated with Nickel-IMAC resin (GE Healthcare) overnight for protein binding. After protein binding and washing, the His-tagged proteins were eluted with elution buffer (0.5 M NaCl, 20 mM sodium phosphate, 300 mM imidazole, pH 6.8). Protein concentrator (Thermo Scientific) was used to exchange the elution buffer with PBS buffer for downstream protein activity test.

### Free Lipoic Acid Detection

The extraction and detection of free lipoic acid using the LC-MS/MS method developed by Chng et al. (2010) with modifications. Equal volume of acetonitrile was added to the supernatant of cell culture or lysate. The mixture was vortex-mixed for 2 min. After cooling at −30^*o*^C for 30 min, the upper phase containing lipoic acid was transferred to a clean tube for evaporation to dryness. The residue was reconstituted with 200 μl of 50% acetonitrile in water. The extracted lipoic acid sample was injected into an LC-MS/MS system (Agilent 1290 liquid chromatograph and Agilent 6550 iFunnel Q-TOF) in negative mode. Chromatographic separation was achieved with an Agilent Eclipse Plus C18 column (2.1 × 100 mm, 1.8 μm, Agilent) at a flow rate of 0.7 ml/min by gradient solution at 0–5.8 min (80–68% A), 5.8–6.5 min (68–15% A) and 6.5–7 min (15–95% A). Mobile phase A is 0.1% acetic acid (pH 4 adjusted with ammonia hydroxide solution) and mobile phase B is acetonitrile. Nebulizer was set at 40 psig, while sheath gas flow rate is 11 l/min. The optimized collision energy for lipoic acid is 8 eV. Quantification was achieved by using 2-propylvaleric Acid (Tokyo Chemical Industry Co., Ltd.) as an internal standard.

Gas chromatography-mass spectrometry (GC-MS) was also used to confirm the identity of lipoic acid. Briefly, HPLC grade ethyl acetate (Sigma) was added to either the supernatant of the cell culture or lysate to extract lipoic acid. The mixture was separated into two phases by centrifugation. The upper phase containing lipoic acid was mixed with N,O-bis(trimethylsilyl)trifluoroacetamide (BSTFA) containing 1% trimethylchlorosilane at a ratio 4:1. The derivatized lipoic acid was analyzed using GC-MS under the following conditions. An HP-5 s column (30 m by 0.25 mm; 0.25 μm film; Agilent) was used with a helium flow rate set to 1 ml/min. Injections of 1 μl were carried out under splitless injection condition with the inlet set to 250^*o*^C. The GC temperature profile was as follows: an initial temperature of 45^*o*^C was maintained for 2 min, followed by ramping to 280^*o*^C at a rate of 10^*o*^C/min, where the temperature was held for 3.5 min. The mass spectrometer detector was scanned from 30 to 800 amu in the electron ionization (EI) mode. To aid peak identification, authentic lipoic acid (Sigma) standard was used as reference.

### Fluorescence Microscopy

*S. cerevisiae* BY4741 cells carrying the plasmids pRS41K-P_GAL1_-EGFP-T_CYC1_ and pRS41K-P_GAL1_-mEGFP-T_CYC1_ were grown to early logarithmic phase in induction medium (YPGR with 200 mg/L G418). The cells were harvested and mounted on a poly-L-lysine-coated glass slide. EGFP fluorescence was visualized with a fluorescent microscope (Leica DMi8).

## Results and Discussion

### Proteomic Analysis and Characterization of Lipoylated Proteins as Substrates for Free Lipoic Acid Biosynthesis

To engineer the yeast for free lipoic acid biosynthesis, we first aimed to evaluate the availability of the various forms of lipoate-bound proteins and understand their formation process. We hypothesized that this would facilitate our selection of a suitable lipoylated protein as substrate for subsequent enzymatically cleavage by EfLPA at the amide linkage to release free lipoic acid. Lipoic acid exists covalently bound to proteins via an amide linkage in *S. cerevisiae*. It was hypothesized that its biosynthesis begins with the transfer of an octanoyl moiety from octanoyl-ACP to the apo form of lipoate-dependent proteins, followed by modification of the octanoyl moiety by insertion of two sulfur atoms (Schonauer et al., 2009). As lipoic acid is mainly bound to three proteins, namely Gcv3p, Lat1p, and Kgd2p, we sought to focus our analysis on these proteins through LC-MS/MS to better our understanding of the protein lipoylation mechanism.

**FIGURE 2:**
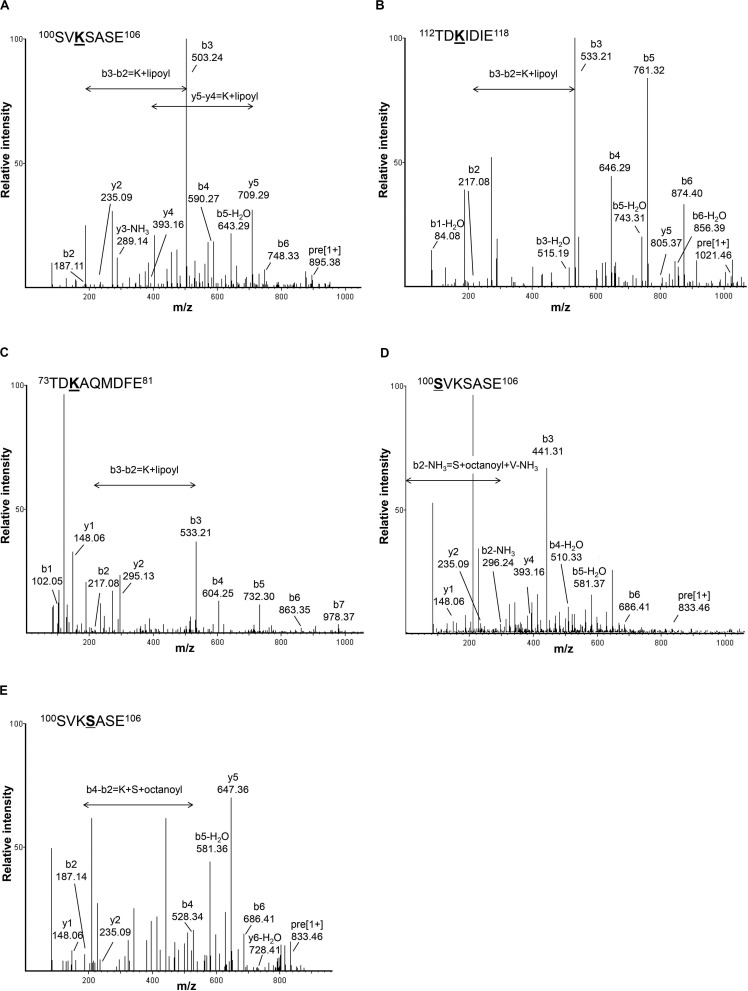
Detection of lipoyl/octanoyl-modified peptides. **(A–C)** show the MS/MS spectra of peptides with lipoic acid modification. Singly charged peptide (m/z = 895.3918) of Gcv3p **(A)** and peptide (m/z = 1021.4584) of Kgd2p **(B)** as well as doubly charged peptide (m/z = 636.7529) of Lat1p **(C)** were detected with lipoic acid modification at position K^102^, K^114^, and K^75^, respectively. **(D,E)** show MS/MS spectra of peptides with octanoic acid modification. Singly charged peptide (m/z = 833.4583) with octanoic acid modification at position S^100^ **(D)** and peptide (m/z = 833.4628) with octanoic acid modification at position S^103^ **(E)** were detected. S, serine; V, valine; K, lysine; A, alanine; E, glutamic acid; T, threonine; D, aspartic acid; I, isoleucine; Q, glutamine; M, methionine; F, phenylalanine; Lipoyl, lipoyl modification; Octanoyl, octanoyl modification.

To investigate the lipoylation of Gcv3p, Lat1p, and Kgd2p, we extracted the total protein from *S. cerevisiae* and separated the proteins into 12 fractions by HPLC with reverse phase column to reduce the complexity of our protein samples. Instead of using trypsin and chymotrypsin reported previously to generate long peptide fragments (Gey et al., 2014), in this study, each protein sample was digested with Glu-C leading to shorter peptides which gives better precision. The digested peptide mixtures were analyzed by LC-MS/MS. In total, 2,713 peptides were identified based on their m/z value and MS/MS spectra. As shown in Figure 2A, a singly charged peptide with m/z 895.3918 was detected. This fragment corresponds to the ^100^SVKSASE^106^ sequence from Gcv3p carrying a lipoic acid modification at K^102^ (lysine^102^). Similarly, a singly charged peptide with m/z 1021.4584 revealed the presence of the sequence ^112^TDKIDIE^118^ from Kgd2p with K^114^ modified by lipoic acid (Figure 2B). A doubly charged lipoylated peptide with m/z 636.7529 detected as a precursor ion indicates that the sequence ^73^TDKAQMDFE^81^ from Lat1p was also modified with lipoic acid at K^75^ (Figure 2C). Therefore, we concluded from our data that Gcv3p, Kgd2p, and Lat1p were lipoylated at positions K^102^, K^114^, and K^75^, respectively, in wild-type cell BY4741. The detailed calculations are shown in Supplementary Figure S1.

**FIGURE 3:**
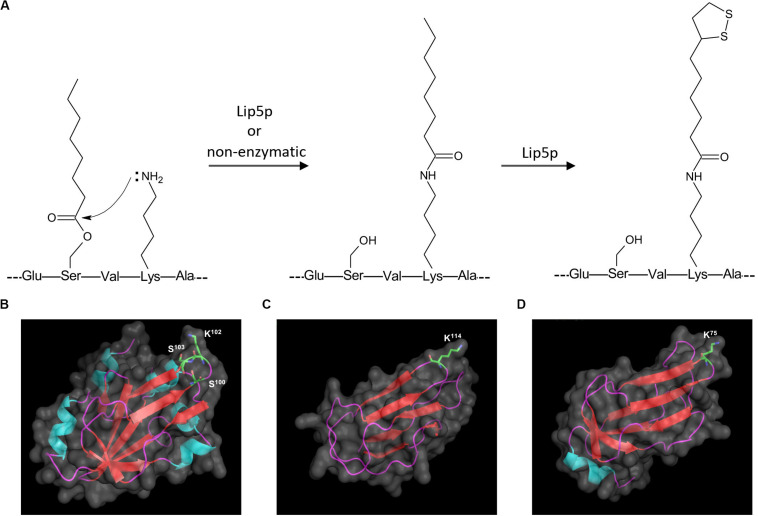
Proposed mechanism for sulfur insertion of Gcv3p **(A)**, and 3D protein structures of **(B)** Gcv3p, **(C)** lipoyl domain of KGD2 and **(D)** lipoyl domain of LAT1. Helix was shown in light blue, sheet in red and loop in purple. Surface of protein was show in gray. K stands for lysine residue while S stands for serine residue.

In addition to lipoylated peptides, we also observed octanoylated peptides in GCV3p that likely originated from precursors of lipoate-proteins. Detection of two singly charged peptides with m/z 833.4583 and 833.4628 indicates single octanoyl modification of the sequence ^100^SVKSASE^106^ at the S^100^ (serine^100^) or S^103^ position, respectively (Figures 2D,E). This suggests that, unexpectedly, binding of lipoate and octanoate does not occur on the same residue but instead takes place on lysine and proximal serine residues, respectively. These data provide the first MS-based evidence of octanoylation of Gcv3p protein at serine residues in the vicinity of the lipoate-modified lysine residue, inferring that Gcv3p is loaded with octanoate at S^100^ or S^103^ to serve as precursors prior to the formation of lipoate-Gcv3p with lipoate-modified K^102^. Therefore, instead of direct octanoylation of lysine followed by addition of sulfur atoms to the octyl carbon chain, we propose that lipoyl-Gcv3p is formed through the following three steps: (i) esterification of the serine (S^100^ or S^103^) side chain with an octanoyl functional group, (ii) amidation of the lysine (K^102^) side chain by acyl transfer of the octanoyl moiety from S^100^ or S^103^ and (iii) insertion of sulfur atoms to the octanoyl moiety by the lipoyl synthase Lip5p (Figure 3A). Interestingly, no octanoylated peptides derived from Kgd2p and Lat1p were detected. One possibility is that octanoylated Kgd2p and Lat1p proteins can be intermediately converted to lipoate-modified protein after they were generated. Alternatively, lipoylation of Kgd2p and Lat1p may occur via amido-transfer from lipoate-Gcv3p, since Gcv3p and Lip3p are essential for forming lipoate-modified Kgd2p and Lat1p, and Lip3p has been suggested to be a possible amidotransferase (Schonauer et al., 2009; Hiltunen et al., 2010).

To elucidate the protein structural characteristics and visualize the locations of the octanoylation and lipolylation sites, we predicted the structures of Gcv3p, Kgd2p, and Lat1p by homology modeling (Figures 3B–D). All the residues for modification, namely K^102^, S^100^ and S^103^ in Gcv3p, K^114^ in Kgd2p, and K^75^ in Lat1p, are positioned on β-turns, which are typically surface-exposed (Marcelino and Gierasch, 2008). Hence, their corresponding octanoyl- and lipoyl-PTMs are present on the protein surface and accessible for enzymatic catalysis to be performed on these residues, i.e., the attachment of octanoic acid to serine residue by Lip2p/Lip3p, the insertion of sulfur atoms to the octanoylated lysine residue by Lip5p and the hydrolysis of the amide bond between the lipoic acid and the lysine residue by EfLPA. Overall, we identified the lysine residues where Gcv3p, Kgd2p, and Lat1p are lipoylated in wild-type BY4741 strain, i.e., K^102^, K^114^, and K^75^, respectively. The discovery of octanoylated serine residues in Gcv3p suggests a lipoylation mechanism whereby octanoylation of the lysine residue involves pre-loading of octanoyl moiety onto serine residues followed by acyl transfer to the lysine side chain. We have also established from the predicted protein structures of Gcv3p, Kgd2p, and Lat1p that their lipoylated lysine residues are accessible to EfLPA for hydrolysis. Hence the activity of EfLPA on lipoylated Gcv3p, Kgd2p, and Lat1p was subsequently characterized to determine the suitability of these lipoylated enzymes as substrates for EfLPA to produce free lipoic acid.

### *In vitro* Characterization of EfLPA for Free Lipoic Acid Biosynthesis

Free lipoic acid is produced by enzymatic cleavage of the amide bond linking the lipoyl moiety to the lysine of lipoate-dependent proteins with a lipoamidase. EfLPA from *E. faecalis* was previously shown to release lipoic acid from lipoate-modified proteins in *E. coli* (Spalding and Prigge, 2009). Lipoic acid is mainly bound to three proteins, namely Gcv3p, Lat1p, and Kgd2p in yeast as demonstrated in Figure 2, but whether EfLPA is functional toward these lipoylated yeast proteins has not been reported. Therefore, to engineer *S. cerevisiae* for free lipoic acid biosynthesis, we characterized the *in vitro* enzyme activity of EfLPA toward these lipoylated proteins. We hypothesized that through this *in vitro* investigation, we could identify a suitable substrate protein candidate that EfLPA is catalytically active on for subsequent overexpression to increase the availability of sites at which lipoic acid can be synthesized.

**FIGURE 4:**
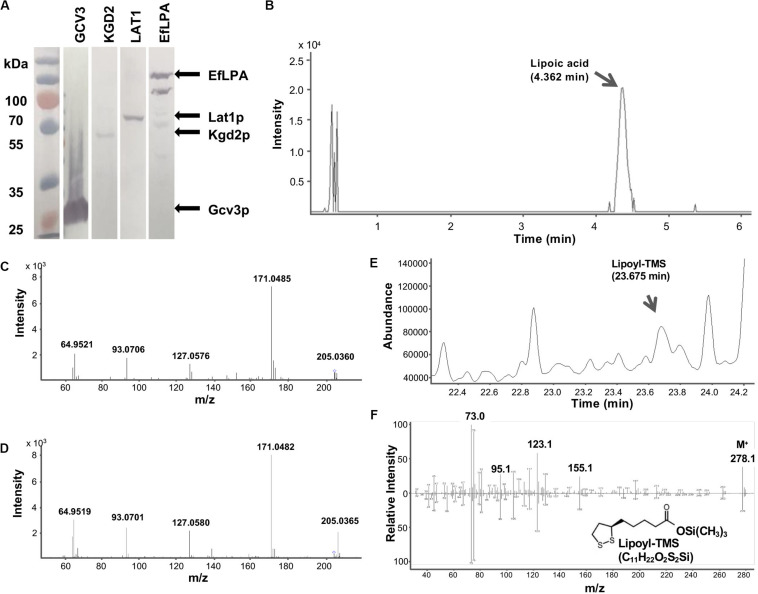
GCV3, KGD2, LAT1 and EfLPA protein expression and lipoamidase activity of EfLPA toward GCV3 *in vitro*. **(A)** Expression of GCV3, KGD2, LAT1 and EfLPA. The expression of GCV3, KGD2, LAT1 and EfLPA were confirmed by western blot analysis. **(B)** LC-MS/MS chromatogram of extracted product from EfLPA and Gcv3p mixture. Peak of lipoic acid is indicated by an arrow at retention time 4.362 min. **(C)** LC-MS/MS spectrum of the single-charged ion of lipoic acid. Lipoic acid detected in **(B)** was further fragmented by MS/MS. **(D)** LC-MS/MS spectrum of the single-charged ion of lipoic acid standard reference. The precursor ions, 205.0360 for **(C)** and 205.0365 for **(D)**, both marked with a diamond. m/z value of product ions were labeled. **(E)** GCMS chromatogram of extract from EfLPA and Gcv3p mixture. Trimethylsilylated lipoic acid (lipoyl-TMS) was detected at a retention time of 23.675 min. **(F)** GCMS spectrum of the lipoyl-TMS peak in **(E)** is shown in the top spectrum. It is identical to the bottom GCMS spectrum obtained using a trimethylsilylated lipoic acid authentic reference standard.

To test the catalytic activity of EfLPA toward lipoylated proteins from yeast, *EfLPA* with hexa-histidine tag was expressed under the strong galactose-inducible P_GAL1_ promoter from a low copy-number plasmid. Lipoate-bound proteins (i.e., Gcv3p, Kgd2p, and Lat1p) fused with a hexa-histidine tag were expressed individually under the strong constitutive promoter P_TEF1_ from the genome. As shown in Figure 4A, the expression of Gcv3p, Kgd2p, Lat1p, and EfLPA in *S. cerevisiae* was confirmed by western blot. Gcv3p showed much higher protein expression than the other proteins, while Kgd2p showed the lowest protein expression. The reason for the low expression levels of Kgd2p and Lat1p is unclear but it has been shown that essential proteins have relatively shorter protein half-lives, which may be due to strict fidelity requirements and lower threshold to damage for essential proteins (Martin-Perez and Villén, 2017). Therefore, the low protein expression of Kgd2p and Lat1p may be due to fast protein turnover since both Kgd2p and Lat1p are involved in aerobic respiration, a central process in cellular metabolism (Schonauer et al., 2009). Western blot analysis of EfLPA protein showed multiple bands, which is consistent with a previous report (Spalding and Prigge, 2009).

To determine whether EfLPA possesses broad-range lipoamidase activity toward lipoylated proteins from yeast, purified Gcv3p, Kgd2p, and Lat1p proteins were incubated with purified EfLPA individually at 37^*o*^C for 2 h. The extracted products from the enzymatic reaction mixtures were analyzed by LC-MS/MS. No lipoic acid was detected in the control reaction mixture containing EfLPA, Gcv3p, Kgd2p, or Lat1p only. Interestingly, no lipoic acid was observed in the reaction mixtures containing EfLPA with Kgd2p or Lat1p individually. Only the reaction of EfLPA with Gcv3p resulted in a peak with m/z 205.0360 (Figure 4B) indicative of lipoic acid. Product ion scan of the abovementioned precursor ion m/z 205.0360 displayed clear and abundant product ions at m/z 64.9521, 93.0706, 127.0576, and 171.0485 (Figure 4C), which is identical to the mass spectrum of a lipoic acid reference standard (Figure 4D). The extracted product was additionally analyzed by GC-MS to further confirm the presence of lipoic acid. Analysis of the trimethylsilyl derivatized product showed a peak with a corresponding mass spectrum identical to that of the reference standard (Figures 4E,F). These results demonstrate that EfLPA has lipoamidase activity toward Gcv3p from yeast *in vitro* and can be potentially used as an amidohydrolase to release free lipoic acid from lipoate-modified proteins in yeast. It is unclear why no lipoic acid was generated by EfLPA from Kgd2p or Lat1p. Structure models of Gcv3p, Kgd2p, and Lat1p show that all the modified residues, i.e., K^102^, S^100^ and S^103^ in Gcv3p, K^114^ in Kgd2p, and K^75^ in Lat1p, are present on β-turns exposed to the solvent on the protein surface, and hence inaccessibility of the lipoylation site is unlikely the reason for the lack of lipoamidase activity of EfLPA on Kgd2p and Lat1p. Other possibilities may be that (i) the protein expression levels of Lat1p and Kgd2p were too low (Figure 4A), (ii) less lipoic acid moiety were attached to Lat1p and Kgd2p proteins compared with Gcv3p (Hermes and Cronan, 2013) or (iii) the substrate specificity of EfLPA excludes both Lat1p and Kgd2p.

Taken together, the *in vitro* results show that Gcv3p, being a better substrate for EfLPA compared to Lat1p and Kgd2p, is the most suitable protein substrate out of the three candidates for subsequent pathway engineering to optimize free lipoic acid biosynthesis. Moreover, Gcv3p is a smaller protein than Kgd2p and Lat1p (19, 50, and 52 kDa, respectively), and thus its overexpression utilizes less resource than the latter proteins. Furthermore, unlike the formation of lipoate-Gcv3p, lipoylation of Kgd2p and Lat1p requires an additional enzyme, i.e., Lip3p, which might reduce the efficiency of lipoylation and increase metabolic burden if *LIP3* overexpression is additionally required. In summary, we established that EfLPA is functionally expressed in *S. cerevisiae* and has activity on Gcv3p, which we therefore selected as the preferred lipoylated protein substrate. These enzymes were employed for subsequent engineering of *S. cerevisiae* to overproduce free lipoic acid *in vivo*.

### Overexpression of EfLPA in the Mitochondria Led to Lipoic Acid Biosynthesis *in vivo*

**FIGURE 5:**
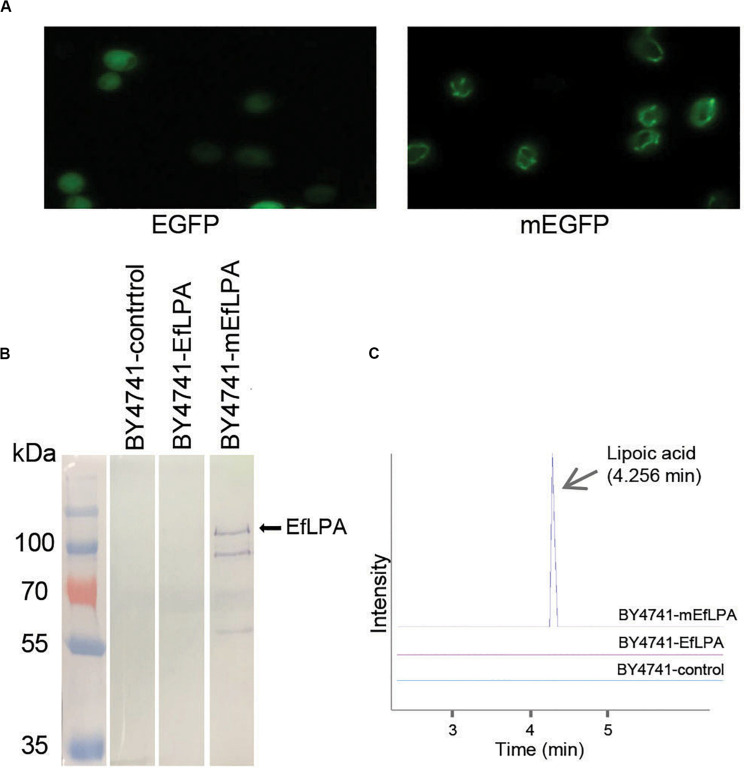
Subcellular localization of EfLPA and lipoic acid production *in vivo*. **(A)** Characterization of the mitochondria targeting peptide. Cells carrying EGFP fused with and without mitochondria signal peptide (mEGFP and EGFP) were harvested. Fluorescence figures were shown. **(B)** Subcellular localization of EfLPA. Proteins in mitochondria of BY4741-control, BY4741-EfLPA and BY4741-mEfLPA cells were extracted. The expression of EfLPA carrying 6xHis tag in mitochondria was confirmed by western blot analysis. **(C)** Lipoic acid production *in vivo*. Lipoic acid was extracted and quantified from BY4741-control, BY4741-EfLPA and BY4741-mEfLPA cells by LC-MS/MS analysis.

As mentioned, lipoic acid synthesis occurs in the mitochondria of yeast. To enable lipoic acid biosynthesis *in vivo*, EfLPA must be translocated to the mitochondria where it hydrolyzes lipoic acid from lipoylated protein substrates. To this end, a 29-amino-acid mitochondrial targeting peptide (MTP) from the yeast cytochrome c oxidase subunit IV (COX4) (Maarse et al., 1984) was explored for translocating proteins to the mitochondria. As shown in Figure 5A, EGFP fused with the MTP was localized in the mitochondria while EGFP without MTP was diffused in the cytosol. To localize EfLPA to mitochondria, EfLPA was fused with the characterized MTP. Mitochondrial proteins were extracted and analyzed by western blotting to determine mitochondrial translocation of EfLPA. Only the extracts from cells expressing MTP-EfLPA fusion protein (mEfLPA) showed a band corresponding to the protein whereas no bands were observed in the extracts from wild-type BY4741 with empty plasmid and cells expressing EfLPA without MTP, hence confirming translocation of EfLPA to the mitochondria when fused with MTP (Figure 5B).

We evaluated the *in vivo* activity of the EfLPA in mitochondria by quantifying the lipoic acid concentrations in cell cultures grown for 3 days. We found that the wild-type BY4741 with empty plasmid and BY4741 expressing *EfLPA* without MTP produced no detectable lipoic acid, whilst the BY4741-mEfLPA strain expressing *EfLPA* in the mitochondria produced free lipoic acid at 10.1 μg/L (Figure 5C). Thus, BY4741-mEfLPA constructed here is the first yeast strain reported with the ability to produce free lipoic acid *in vivo* and served as the base strain for further engineering to improve titer.

### Expression of Pathway Enzymes and Regeneration of Cofactor Improved Lipoic Acid Production

**FIGURE 6:**
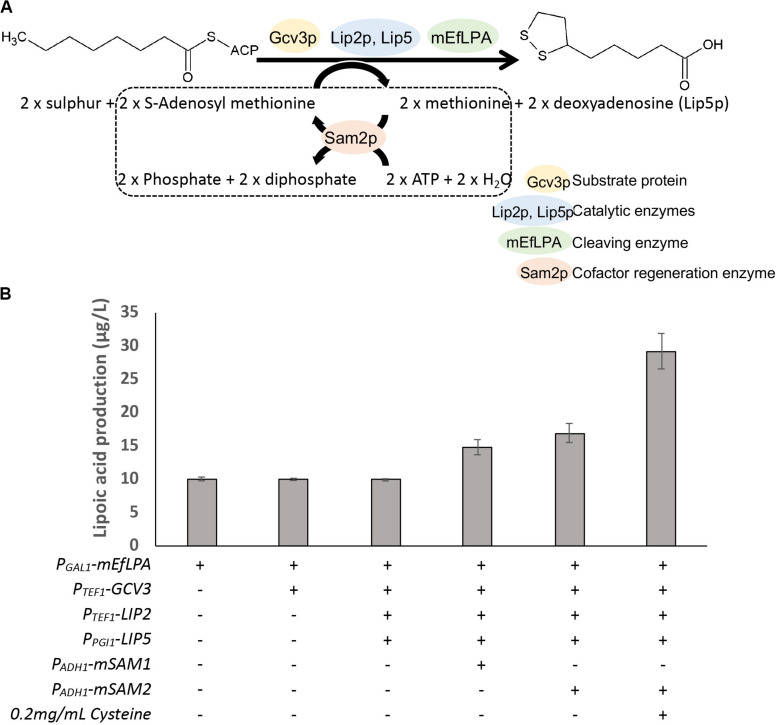
Production of lipoic acid using different engineered strains. **(A)** The overall pathway engineering for lipoic acid production. Dotted box represents the cofactor regeneration reaction catalyzed by Sam2p. **(B)** The comparison of total lipoic acid produced by the expression of different enzymes. “+” and “-” indicate presence and absence of the respective modifications. Data shown are the mean ± SD of three biological replicates.

The overall genetic engineering for lipoic acid production *in vivo* is shown in Figure 6A. As a first step to improve lipoic acid production, we attempted to increase the availability of lipoylation sites by overexpressing a suitable protein candidate such that more lipoylated proteins can form to serve as substrates for EfLPA hydrolysis. Specifically, as determined in section “*In vitro* Characterization of EfLPA for Free Lipoic acid Biosynthesis,” GCV3p was selected to be the protein candidate for overexpression. To this end, we co-expressed *GCV3* under P_TEF1_ from the genome along with *mEfLPA*, hence generating the strain BY4741-GCV3-mEfLPA. However, as shown in Figure 6B, overexpression of GCV3p did not improve free lipoic acid production. This suggests that the bottleneck in free lipoic acid production from strain BY4741-mEfLPA is not the inadequacy of substrate protein, which can be recycled during free lipoic acid production, but possibly insufficient activity of the catalytic enzymes and/or cofactors required to synthesize the lipoyl moiety (Figure 1).

The catalytic enzyme Lip2p, an octanoyltransferase, has been demonstrated to convert apo-Gcv3p to octanoyl-Gcv3p while another catalytic enzyme Lip5p, a lipoyl synthase, catalyzes the conversion of octanoyl-Gcv3p to lipoyl-Gcv3p (Hermes and Cronan, 2013; Figure 1). Thus, to increase the level of lipoyl-Gcv3p, *LIP2* was expressed under the strong P_TEF1_ promoter while *LIP5* was expressed under the weak P_PGI1_ promoter (as expression of *LIP5* under the strong P_TEF1_ promoter caused cell inviability). However, the resulting strain overexpressing *GCV3*, *LIP2*, *LIP5*, and *mEfLPA* showed similar lipoic acid production compared with cells expressing *mEfLPA* only (Figure 6B), suggesting that the activities of Lip2p and Lip5p are not rate-limiting for lipoic acid production.

Another possible rate-limiting factor for lipoic acid production in yeast is the availability of cofactors, particularly S-adenosylmethionine (SAM), which is required for sulfurization of the octanoyl moiety. Homologous lipoyl synthase from *E. coli* uses radical SAM chemistry to perform the insertion of two sulfurs into the octanoyl moiety, a process that requires both the cofactor SAM and the iron-sulfur clusters in the lipoyl synthase (Cicchillo et al., 2004). Radical intermediates are generated from SAM to abstract hydrogen atoms from C-6 and C-8 of the octanoyl moiety, allowing for subsequent sulfur insertion by a mechanism involving carbon-centered radicals. Iron-sulfur cluster in the lipoyl synthase provides an electron during the cleavage of SAM for radical generation and also may act as the source for sulfur atoms during lipoylation (Cicchillo and Booker, 2005). Therefore, increasing the availability of SAM and functional iron-sulfur clusters may drive the formation of lipoyl moiety. In *S. cerevisiae*, SAM can be generated from methionine and ATP by the lipoyl synthases Sam1p and Sam2p (Marobbio et al., 2003; Dato et al., 2014). To increase SAM availability by regeneration from methionine and ATP, *SAM1*, and *SAM2* were fused with MTP for mitochondria translocation and overexpressed under the weak P_ADH1_ promoter. Overexpression of the mitochondrial *mSAM1* or *mSAM2* increased lipoic acid production to 14.8, and 17.0 μg/L, respectively (Figure 6B), suggesting that SAM availability is a critical bottleneck in lipoic acid production. To form the iron-sulfur clusters in the lipoyl synthases, ferrous ions need to be imported from the medium and sulfur has to be released from cysteine through the iron-sulfur cluster assembly machinery (Lill et al., 2006). Therefore, to further drive the synthesis of the lipoyl moiety, the cell culture of the highest lipoic acid producer, i.e., the strain overexpressing *GCV3*, *LIP2*, *LIP5*, *mSAM2*, and *mEfLPA*, was supplemented with ferrous sulfate and cysteine, which can be transported into mitochondria (Philpott and Protchenko, 2008; Lee et al., 2014). Addition of ferrous sulfate was not beneficial for lipoic acid production (11.3 μg/L). In contrast, supplementation with cysteine increased lipoic acid production to 29.2 μg/L, representing almost 2.9-fold increase in titer over that from the base strain BY4741-mEfLPA. This result suggests that cysteine provides sulfur for iron-sulfur cluster biogenesis and utilization by the lipoyl synthase Lip5p to insert sulfur atoms into the carbon chain of the octanoyl group.

While we have identified a few rate-limiting steps in the lipoic acid production pathway, there is still much space for improvement to enhance lipoic acid production. To further boost the titer of lipoic acid, ion-sulfur cluster biogenesis and SAM availability, which are limiting factors of lipoic acid bio-production, can further be engineered in the future. In addition, to generate a molecule of lipoic acid, a molar equivalent of the precursor octanoyl-ACP is required (Figure 6A). Therefore, methods to increase octanoyl-ACP supply can be explored to improve lipoic acid production. Moreover, since all the reactions take place in the mitochondria, strain engineering to increase the population of the organelle (Visser et al., 1995) can be another potential approach to increase lipoic acid titer (Zhou et al., 2016). More studies are needed to resolve the bottlenecks in the lipoic acid biosynthesis pathway to markedly increase the production level. Further improvement in lipoic acid biosynthesis in yeast may be accelerated in future with rapid advances in synthetic biology and synthetic genomics for *S. cerevisiae*, which will offer novel tools for engineering yeast to acquire beneficial characteristics and serve as superior microbial cells factories (Chen et al., 2018; Foo and Chang, 2018; Xia et al., 2019).

## Conclusion

In this study, we aimed to develop a bio-based method for environmentally friendly lipoic acid production by metabolic engineering of *S. cerevisiae*. To achieve this goal, we sought to (i) understand the lipoylation process in *S. cerevisiae*, (ii) characterize the function of EfLPA toward lipoylated proteins from yeast, (iii) employ EfLPA to enable *S. cerevisiae* to produce free lipoic acid *in vivo* and (iv) improve lipoic acid production using metabolic engineering strategies. We first confirmed the presence of protein-bound lipoate through LC-MS/MS. Using homology modeling techniques, the protein structure of Gcv3p, Kgd2p, and Lat1p were predicted and the residues for modification were found to be solvent-exposed, and hence accessible to enzymes acting on these residues. Through *in vitro* activity analysis, EfLPA was validated to release lipoic acid from yeast lipoyl-Gcv3p, hence demonstrating the first reported functional expression of EfLPA in yeast for releasing lipoic acid from lipoate-bound yeast protein. Subsequently, overexpression of EfLPA in the mitochondria led to lipoic acid production *in vivo*, thus accomplishing unprecedented free lipoic acid biosynthesis in the yeast *S. cerevisiae*. To enhance lipoic acid production, metabolic engineering approaches, including overexpression of pathway enzymes and regeneration of cofactors, were employed and the titer of lipoic acid production in *S. cerevisiae* was boosted by nearly 2.9-fold to 29.2 μg/L. Collectively, the protein analysis, enzyme characterization, structure modeling and combinatorial metabolic engineering approaches in this study provided a better understanding of the lipoic acid production pathway and revealed strategies to improve it. We envisage that the knowledge gained from this study will provide insights on lipoic acid biosynthesis in *S. cerevisiae* and spearhead future efforts in lipoic acid production in yeast.

## Data Availability Statement

All datasets presented in this study are included in the article/Supplementary Material.

## Author Contributions

BC performed the experiments and analyzed experimental data. JF, HL, and MC oversaw the project and provided guidance. BC, JF, and MC wrote, reviewed, and edited the manuscript. All authors have read and agreed to the published version of the manuscript.

## Conflict of Interest

The authors declare that the research was conducted in the absence of any commercial or financial relationships that could be construed as a potential conflict of interest.

## Footnotes

1 http://www.uniprot.org/statistics/Swiss-Prot

2 http://swissmodel.expasy.org/
