# Supplementary material for: Mechanism-Driven Metabolic Engineering for Bio-Based Production of Free R-Lipoic Acid in Saccharomyces cerevisiae Mitochondria
Source: Front Bioeng Biotechnol. 2020 Aug 20;8:965. doi: 10.3389/fbioe.2020.00965 (PMC7468506; doi:10.3389/fbioe.2020.00965)
Supplement: Supplementary file 1 [file Image_1.PDF]

A ^{100}SV**K**SASE^{106}

#	b	b-H2O	b-NH3	b (2+)	Seq	y	y-H2O	y-NH3	y (2+)	#
1	88.04	70.03	71.01	44.52	S					7
2	187.11	169.10	170.08	94.05	V	808.36	790.35	791.33	404.68	6
3	503.24	485.23	486.21	252.12	K(+188.03)	709.29	691.27	692.28	355.14	5
4	590.27	572.26	573.24	295.63	S	393.16	375.15	376.17	197.08	4
5	661.31	643.29	644.28	331.15	A	306.13	288.12	289.14	153.56	3
6	748.33	730.32	731.31	374.67	S	235.09	217.08	218.07	118.05	2
7					E	148.06	130.05	131.03	74.53	1

B ^{112}TD**K**IDIE^{118}

#	b	b-H2O	b-NH3	b (2+)	Seq	y	y-H2O	y-NH3	y (2+)	#
1	102.06	84.08	85.03	51.53	T					7
2	217.08	199.07	200.06	109.04	D	920.41	902.40	903.38	460.71	6
3	533.21	515.19	516.18	267.11	K(+188.03)	805.37	787.37	788.36	403.19	5
4	646.29	628.28	629.27	323.65	I	489.26	471.24	472.23	245.13	4
5	761.32	743.31	744.29	381.16	D	376.17	358.16	359.14	188.59	3
6	874.40	856.39	857.40	437.70	I	261.14	243.13	244.12	131.07	2
7					E	148.06	130.05	131.03	74.53	1

C ^{73}TD**K**AQMDFE^{81}

#	b	b-H2O	b-NH3	b (2+)	Seq	y	y-H2O	y-NH3	y (2+)	#
1	102.05	84.04	85.03	51.53	T					9
2	217.08	199.07	200.10	109.04	D	1171.45	1153.44	1154.42	586.23	8
3	533.21	515.20	516.23	267.11	K(+188.03)	1056.42	1038.41	1039.39	528.71	7
4	604.25	586.23	587.22	302.62	A	740.29	722.28	723.26	370.65	6
5	732.30	714.29	715.28	366.66	Q	669.28	651.24	652.23	335.13	5
6	863.35	845.34	846.32	432.16	M	541.19	523.18	524.17	271.10	4
7	978.37	960.35	961.36	489.69	D	410.15	392.15	393.13	205.58	3
8	1125.44	1107.43	1108.41	563.22	F	295.13	277.12	278.10	148.06	2
9					E	148.06	130.05	131.03	74.53	1

D ^{100}**S**VKSASE^{106}

#	b	b-H2O	b-NH3	b (2+)	Seq	y	y-H2O	y-NH3	y (2+)	#
1	214.14	196.13	197.12	107.57	S(+126.10)					7
2	313.21	295.20	296.24	157.11	V	620.32	602.31	603.30	310.66	6
3	441.30	423.29	424.28	221.15	K	521.26	503.25	504.23	261.13	5
4	528.34	510.33	511.31	264.67	S	393.16	375.15	376.13	197.08	4
5	599.37	581.36	582.39	300.19	A	306.13	288.12	289.10	153.56	3
6	686.41	668.40	669.40	343.70	S	235.09	217.08	218.07	118.05	2
7					E	148.06	130.05	131.03	74.53	1

E ^{100}SVK**S**ASE^{106}

#	b	b-H2O	b-NH3	b (2+)	Seq	y	y-H2O	y-NH3	y (2+)	#
1	88.04	70.03	71.01	44.52	S					7
2	187.14	169.10	170.08	94.05	V	746.43	728.41	729.40	373.71	6
3	315.20	297.19	298.18	158.10	K	647.36	629.35	630.35	324.18	5
4	528.34	510.33	511.31	264.67	S(+126.10)	519.27	501.26	502.24	260.13	4
5	599.37	581.36	582.37	300.19	A	306.13	288.12	289.10	153.56	3
6	686.41	668.40	669.41	343.70	S	235.09	217.08	218.07	118.05	2
7					E	148.06	130.05	131.03	74.53	1

Supplementary Figure 1. Detection of product ions (b and y) in lipoyl/octanoyl-modified peptides. (A)-(C) show the calculated m/z of ions in MS/MS spectra of peptides with lipoic acid modification. **(D)-(E)** show the calculated m/z of ions in MS/MS spectra of peptides with octanoic acid modification. The sequences obtained are shown at the top of the table. Detected b ions are shown in blue while detected y ions are shown in red. "#" indicates the position of the amino acid in the sequence. 188.03 and 126.10 represent the masses of lipoyl and octanoyl, respectively.
