# Supplementary material for: Mechanism-Driven Metabolic Engineering for Bio-Based Production of Free R-Lipoic Acid in Saccharomyces cerevisiae Mitochondria
Source: Front Bioeng Biotechnol. 2020 Aug 20;8:965. doi: 10.3389/fbioe.2020.00965 (PMC7468506; doi:10.3389/fbioe.2020.00965)
Supplement: Supplementary file 2 [file Table_1.PDF]

Table S1: Primers used in this study. Restriction sites are in bold.

Primers	Primer sequences (5′–3′)
P_{GAL1}-F	AAAC**GAGCTC**AGTACGGATTAGAAGCC
P_{GAL1}-R	TTTTTAGGGTTTTTTCTCCTTGACGTT
T_{CYC1}-F	ATCCGCTCTAACCGAAAAGG
T_{CYC1}-R	AAAC**GAGCTC**CTTCGAGCGTCCCAAAACC
EfLPA-F	CGTCAAGGAGAAAAAACCCTAAAAAATGCTAGCCCAAGAA
mEfLPA-F	CGTCAAGGAGAAAAAACCCTAAAAAATGCTTTCACTACGTCAATCTATAAGATTTTTCAAGCCAGCCACAAGAACTTTGTGT AGCTCTAGATATCTGCTTCAGCAAAAACCCATGCTAGCCCAAGAA
EfLPA-R	CTAACTCCTTCCTTTTCGGTTAGAGCGGATTCATTAATGGTGATGGTGATGATGCTTACGGGTCTTTCTAATGTAGA
EGFP-F	CGTCAAGGAGAAAAAACCCTAAAAAATGTCTAAAGGTGAA
mEGFP-F	CGTCAAGGAGAAAAAACCCTAAAAAATGCTTTCACTACGTCAATCTATAAGATTTTTCAAGCCAGCCACAAGAACTTTGTGT AGCTCTAGATATCTGCTTCAGCAAAAACCCATGTCTAAAGGTGAA
EGFP-R	CTAACTCCTTCCTTTTCGGTTAGAGCGGATTCATTAATGGTGATGGTGATGATGTTTGTACAATTCATC
P_{TEF1}-F	ACCG**CTCGAG**CATAGCTTCAAAATGTTTCTACTCCTTT
P_{TEF1}-R	TTGTAATTAAAACTTAGATTAGATTGC
GCV3-F	GCAATCTAATCTAAGTTTTAATTACAAATGTTACGCACTACTAGACTATGG
GCV3-R	CTAACTCCTTCCTTTTCGGTTAGAGCGGATTCATTAATGGTGATGGTGATGATGGTCATCATGAACCAGTGT
KGD2-F	GCAATCTAATCTAAGTTTTAATTACAAATGCTTTCCAGAGCGACG
KGD2-R	ATCAGATTGGTATGGGCTGCAAATTTCAAATCATTAATGGTGATGGTGATGATGCCATAACAACATTTTTCTAG
T_{KGD2}-F	TTTGAAATTTGCAGCCCATAC
T_{KGD2}-R	ATTC**GAGCTC**ATGTGGAAATCAAAAGAATATTAGTTGAT
LAT1-F	GCAATCTAATCTAAGTTTTAATTACAAATGTCTGCCTTTGTCAGGGTG
LAT1-R	TAATAAAAATCATAAATCATAAGAAATTCGTCATTAATGGTGATGGTGATGATGCAATAGCATTTCCAAAGGAT
T_{ADH1}-F	CGAATTTCTTATGATTTATGATTTTTA
T_{ADH1}-R	ACGC**GGATCC**GAGCGACCTCATGCTATACCT
LIP2-LIP5-CS6-F	AACCTCGAGGAGAAGTTTTTTTACCCCTCTCCACAGATC**CTCGAG**CATAGCTTCAAAATGTTTCTAC
LIP2-LIP5-CS6-R	TAATTAGGTAGACCGGGTAGATTTTTCCGTAACCTTGGTGTC**GAGCTC**ACGCATTTTTTTCTTTTGC
SAM1/2-CS8-F	CAAAATTACCTACGGTAATTAGTGAAAGGCCAAAATCTAATGTTACAATAGTATACTAGAAGAATGAGCCAAG
SAM1-CS8-R	GACCGTTCCCTTGTGTTGTACCAGTGGTAGGGTTCTTCTCGGTAGCTTCTATAAGATAAAGTTTGGTTTGTTGATC
SAM2-CS8-R	GACCGTTCCCTTGTGTTGTACCAGTGGTAGGGTTCTTCTCGGTAGCTTCTCCTCAAAGACATTCTATATTTCAACC
